# The association between obesity and depression in adults: a meta-review

**DOI:** 10.1192/bjo.2021.712

**Published:** 2021-06-18

**Authors:** Abdi Malik Musa, Samuele Cortese, Olivia Bloodworth

**Affiliations:** University of Southampton

## Abstract

**Aims:**

Obesity and depression are increasing in prevalence and have become key issues in the public health of the modern day. We performed a meta-review to summarise the association between obesity and depression in adults.

**Method:**

A systematic literature search was undertaken on MEDLINE, PsychINFO, EMBASE and Web of Science for systematic reviews (SRs) with or without meta-analyses (MA) on the association between obesity and depression in adults (>18 years) published before 18 September 2018. Any approach to define depressive disorders (e.g. via structured interview or code in medical file) was accepted. Likewise, any method to assess obesity was accepted. Screening, data extraction and quality assessment was completed by two reviewers independently, with a third reviewer to arbitrate any disagreement. AMSTAR 2 tool was used to assess the methodological quality and risk of bias of the pertinent SRs/MAs.

**Result:**

After duplicate removal, we identified 6007 potentially pertinent citations. Following, title, abstract and full-text screening, 10 studies were included in the review; nine SRs with MAs and one SR. A statistically significant association between obesity and depression was reported in all nine SRs with MAs, with odds ratios ranging from 1.18 (95% CI = 1.11-1.26) to 1.57 (95% CI = 1.53-2.01). Increased severity of obesity (body mass index over 40) was associated with a greater odds of becoming depressed. Odds of developing depression were greater for obese females, compared to obese males, but this difference was not statistically significant. Depression was shown to be a significant risk factor for future obesity in all four relevant MAs with odds ratios ranging from 1.18 (95% CI = 1.13-1.23) to 1.40 (95% CI = 1.14-1.71) . Depressed adolescent females had the highest odds of becoming obese, significantly more so than depressed adolescent males and depressed adults. The quality of the included studies were mixed with five scoring moderate quality, three low quality and two critically low quality.

**Conclusion:**

The findings suggest a reciprocal association between depression and obesity, which may be modulated by age and gender. Future research should assess the potential effect of obesity and depression severity more carefully while also exploring the underlying mechanisms. These results warrant the investigation of the effect of obesity or depression intervention on the outcomes of the other.

FUNDING

This research received no financial sponsorship.

